# Perception of Acceptance and Discrimination Among the LGBTQI + Community in their Churches and its Association with Spiritual Dryness: Findings from a Cross-Sectional Study in Germany

**DOI:** 10.1007/s10943-024-02023-6

**Published:** 2024-03-22

**Authors:** Arndt Büssing, Lorethy Starck, Klaus van Treeck, Traugott Roser

**Affiliations:** 1 Professorship Quality of Life, Spirituality and Coping, Faculty of Health, Witten/Herdecke University, Gerhard-Kienle-Weg 4, 58313 Herdecke, Germany; 2 Institut für ganzheitliches Wohlbefinden, Resilienz und Spiritualität. Ein AnInstitut der Theologischen Hochschule Friedensau, 28215 Bremen, Germany; 3 Professorship Practical Theology, Faculty of Theology, University of Münster, 48143 Münster, Germany

**Keywords:** Acceptance and discrimination, LGBTQI+, Psychological wellness, Loss of faith, Spiritual dryness

## Abstract

Data from a cross-sectional survey with options for free text statements revealed that people who identify themselves as part of the LGBTQI+ community (*n* = 417) experienced both acceptance and discrimination by church members. Their negative experiences affected their relationship with God in terms of spiritual dryness and loss of faith. In regression analyses, the best predictors of life satisfaction and psychological well-being were self-acceptance and low spiritual dryness. This self-acceptance as a resource, mediated the link between spiritual dryness and life satisfaction. Nevertheless, 96% still wish for a church/faith community that welcomes all people—and accepts them as they are and feel.

## Introduction

Humanity, compassion, empathy, cooperation, and love are fundamental interpersonal experiences of social connectedness (Bauer, 2007). Social acceptance and attachment are essential for holistic well-being, as well as in gender-diverse groups (Romani et al., 2021). However, gender-diverse people experience more symptoms of depression and anxiety than binary people, and these symptoms are mediated by perceived discrimination and humiliation (Meyer, 2003; Romani et al., 2021). Being accepted by others also relates to self-acceptance. Particularly in LGBQ + people,^1^ low self-acceptance is considered a risk factor for mental distress and low psychological well-being (Camp et al., 2020). Being constantly confronted with negative views and statements in their religious communities may result in feelings of shame, guilt, and repression (Ritter & O’Neill, 1989). These negative perceptions of shamefulness, insecurity, anxiety, and self-projected hatred may arise from being socialized with a majority society’s norms and values they cannot apply to themselves, as suggested by Wierz and Nürnberg (2023).

Discrimination is experienced in multiple forms and situations, as well as in church communities. Accepting people with other than a heteronormative orientation is a challenging topic in all religious traditions (Barringer, 2020; Itzhaky & Kissil, 2015; Jaspal & Cinnirella, 2010; Jung, 2021; Lapinski & McKirnan, 2013; Rodriguez, 2010). It is controversially discussed in almost all churches and their local congregations (Deeg, 2020; Roser, 2015). While churches often seem to take a coherent position on non-marital sexual behavior, sexual orientation, or gender identity, their changing attitudes and—over time—contradictory regulations demonstrate the diverse nature and imperfections and also distinct forms of acceptance and acceptability of members of the LGB community. Although the aim is not to give a comprehensive overview of the different positions of the distinct church communities (Pew Research Center, 2012), some churches and their officials see the heterosexual marriage of man and woman as the sole form of a biblically based marriage (Adventist Family Ministries, 2012; American Baptist Churches USA, 2005; Texas Baptists, 2023, Catechism of the Catholic Church 1997; Evangelische Kirche in Deutschland, 1996), while other churches allow LGBTQI+ marriage or at least same-sex blessings (Bubmann et al., 2017; Evangelical Lutheran Church in America, 2009; Roser, 2022). Conservative Christian protagonists often rely on a literal interpretation of biblical texts without regard to their specific cultural contexts, while others disagree with these literal interpretations (Johnston, 2022; Salzmann & Lawler, 2020; Schmitt, 2023). Today, the Roman Catholic Church regards a homosexual *orientation* not as sinful per se, while homosexual sexual *activity* is regarded as "contrary to the natural law" (Catechism of the Catholic Church, 1997). Despite several recommendations or statements, there is an ongoing, often very emotional discussion about this topic. Mainline protestant churches in Germany and other European countries have differed vastly in their dealings with homosexuality and queer persons, especially with clergy who consider themselves part of the LGBTQI+ community (Hancocks et al., 2008; Roser, 2022; Söderblom, 2023). The 2023 Statement of the German Unions of Seventh-Day Adventists (SDA) argues that the “creation story outlines an image of sexual identity and intimacy that has its place only within the marital relationship between a man and a woman” and that “the acceptance of LGBTQ+ persons opens up an area of tension with biblical texts” (Freikirche der Siebenten-Tags-Adventisten, 2023). Nevertheless, they also stated that “our personal beliefs should never be an obstacle to loving and accepting others, even if our own beliefs are different,” and that there is a demand to “listen to our fellow human being and perceive him or her as a person loved by God” (Freikirche der Siebenten-Tags-Adventisten, 2023).

In light of the discussions on this topic and some changes within the general society (Keleher & Smith, 2012), including a shift from the registered partnership in 2001 to legalizing same-sex marriages in 2017 in Germany, and the approval of blessings of same-sex relationships by the Vatican in December 2023 (Pullella, 2023): Do people who consider themselves part of the LGBTQI+ community feel welcome and comfortable in their church or parish, and how do they feel about faith, religion, and spirituality?

In this empirical study, we intended to give people who belong to the LGBTQI+ community and who are still trying to live as part of their church community a voice. Several of them feel deeply hurt by strict positions against their sexual orientation and gender identity. Often enough, they cannot or do not even want to talk about it anymore, terminate their church membership, and leave the church. This research project should thus be seen in the context of continuing research (Barret & Barzan, 1996; Barringer, 2020; Ritter & O’Neill, 1989; Rodrigues, 2010; Romani et al., 2021) on how genderqueer and non-binary people who may have an active religious life feel accepted within their church, following an argument of Rodriguez (2010) that queer and non-binary people should be seen as “spiritual and religious beings in their own right, rather than merely sexual beings needing to be compared and contrasted with religious others” (p. 5).

To explain the higher risk of LGBs for mental disorders than heterosexuals, Meyer (2003) attributed these observations to the concept of minority stress where “stigma, prejudice, and discrimination create a hostile and stressful social environment that causes mental health problems” (p. 674). The concept of minority stress as a factor for increased risk for mental health among gay, lesbian, and bisexual persons in Germany has been supported by Sattler (2018), too. Important findings came from Saunders et al. (2023) who argued that sexual minorities were stigmatized by religious groups and institutions for years and that this could be related to an increased risk of depressive mood states during the process of transition to adulthood. In their study, LGB people who became affiliated with a religious group had more depressive symptoms than consistently unaffiliated people; overall, being constantly unaffiliated was less harmful to their mental health (Saunders et al., 2023).

In this study, two aspects relating to LGBTQI+ peoples´ sexual orientation (and their identity) are in the foreground: (a) the experience of acceptance or exclusion in the church communities and (b) one's attitude toward oneself and one's identity. These aspects will be referred to as life satisfaction and psychological well-being, on the one hand, and to the perception of spiritual dryness, which is a specific form of crisis related to the perception of God (Büssing, 2013; Büssing & Dienberg, 2019, 2021), on the other hand. It is assumed that the experience of acceptance by others (as a horizontal level of relationship) can also influence the experience toward God (as a vertical level of relationship) (Büssing, 2013; Büssing & Dienberg, 2019, 2021). Findings from Lapinsky and McKiernan (2013) showed that people with an LGB identity who have left their church “viewed God as hostile.” The main reason might be seen in the emotional reactions toward the negative responses by the religious community/church which is transferred to their view of God. Even when one may be less satisfied with the support of the church community, one may be satisfied with the support from close friends that could counteract the aforementioned stressor. All this could be conceptualized as “fractured relationships,” as supposed by Exline (2002). She argued that in some situations, interpersonal strains may result in distress perceptions that finally may weaken religious commitment and can negatively influence attitudes toward God (Exline (2002)). Fractured relationships apply also to LGB clergy. Hancocks et al. (2008) described that for several homosexual health-care chaplains in England, their negative experience of the institutional Church and—to a lesser degree—of a parish led them to leave parish clergy and seek employment with the secular national health system. The authors stated that “there is an irony that in order to live in stable relationships clergy have sought safety in secular organizations” (Hancocks et al., 2008; p. 177). A further theoretical concept that could be referred to is the strain–struggles–distress model by Hill et al. (2017) which assumes that stressful experiences, including discrimination, can lead to religious struggles, turning away from God and losing faith “when they can no longer make coherent religious sense of their lives” (Hill et al., 2024; p. 204). They found that the perception of discrimination (i.e., being disrespected, insulted, and harassed) is related to religious struggles and depressive symptoms, and concluded that these struggles could be regarded as a “maladaptive coping response to discrimination” (Hill et al., 2017).

A protective variable against perceived discrimination in the church could be self-acceptance. This resource is regarded as a subjective awareness that accepts all personal attributes, not only the positive but also the conflicting ones (MacInnes, 2006; Shepard, 1979). As a matter of inner strength, it protects against negative statements. Self-acceptance and positive relationships are relevant contributors to psychological well-being (Ryan et al., 2008). Particularly in LGBQ+ people, low self-acceptance is considered a risk factor for mental distress and low psychological well-being (Camp et al., 2020). In their systematic review, Camp et al. (2020) underlined that LGBQ + individuals had “lower general self-acceptance compared to heterosexual participants” (p. 2353).

**Fig. 1:**
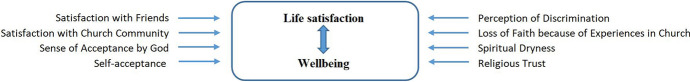
Theoretical model of positive and negative influences on life satisfaction and well-being.

At the forefront of this explorative study is the acceptance by the local church community and the perceived discrimination within the church, which may result in struggles with God (as indicated by spiritual dryness) and maybe loss of faith. The underlying processes may finally have a negative impact on both psychological well-being and life satisfaction in general. The related variables were set in a working model as indicated in Fig. 1:

1. Satisfaction with friends and with the local church community
2. Perception of discrimination and loss of faith because of negative experiences in the church
3. Self-acceptance and sense of acceptance by God
4. Religious trust (“Faith as a firm hold”) and spiritual dryness

These variables will be tested in a cross-sectional survey among German-language people who identify themselves as part of the LGBTQI + community.

## Materials and Methods

In the following, enrolled participants and standardized measures used in this cross-sectional study were described.

### Participants

Participants who identify themselves as part of the LGBTQI+ community were invited by email via religious communities, support groups, and specific networks, and were encouraged to forward the information about this anonymous survey (“snowball sampling”). It is unclear whether all of the target groups could be reached due to the sensitive topic and the recruitment channels.

Since the respondents are a vulnerable group in the sense of a socially constructed, structural disadvantage, a vote was obtained from the ethics committee of the University of Witten/Herdecke (#264/2022). After additional feedback from individuals in the community, the wording on gender identity and sexual orientation (GI/SO) was adjusted. Furthermore, we consulted an employee of the German Society for Transidentity and Intersexuality dgti e.V.

By clicking the tick box (Yes / No), participants gave informed consent that they had read the information about the project and data protection (i.e., anonymous data processing and no recording of IP addresses), and that by completing and sending the questionnaire, they declare their consent to participate and to the anonymous processing of their statements.

Recruitment started on November 25, 2022, and ended on January 26, 2023, in German-speaking countries (84% Germany, 13% Switzerland, and 3% from Austria).

### Measures

The questionnaire asks for sociodemographic data and religious affiliation. The two largest churches in Germany are the Roman Catholic Church (20.9 million members) and the Protestant Churches of Germany (the federation of 20 independent Lutheran, Reformed, and United regional churches with 19 million members). Membership is granted by baptism and is often based on family tradition. The approximately 15 Protestant Free Churches in Germany with voluntary membership, organizational independence, and certain theological attitudes have around 280,000 members.

As some of the participants may identify themselves as part of the LGBTQI+ community because of their sexual orientation and others by their gender identity, both were recorded separately as gender identity (GI: female, male, genderqueer/non-binary, and trans*) and sexual orientation (SO: heterosexual, bisexual, pansexual, homosexual, and other). Here, participants´ assignments and feelings are in the foreground. Their partner status was categorized as living with or living without a permanent partner (i.e., single, divorced, and widowed).

#### Perceptions of Acceptance and Discrimination

With 12 items, we differentiated three perspectives: (1) acceptance (or rejection) by church members, (2) sense of acceptance by God, and (3) self-acceptance (see Table 3). Participants were asked for their perceptions of being accepted or rejected because of their GI/SO, feeling uncomfortable in their community because of their GI/SO, etc. As a consequence of these perceptions, they may have left the church or have lost their faith (item a0: “I don't believe in God anymore”). The five acceptance and discrimination items (Cronbach´s alpha = 0.69) that explain 68% of the variance, differentiate into two sub-constructs, (1) perception of discrimination (items z1 and z2: Cronbach´s alpha = 0.75) and (2) loss of faith because of experiences in church (items z6, z8, a0: Cronbach´s alpha = 0.62).

Also, positive views were addressed (item a1: “I am sure that God loves and accepts me just the way I am”) and two self-acceptance items (items r10: “I have found a positive attitude towards myself”; r1: “All in all, I'm happy with myself”; Cronbach´s alpha = 0.87).

Finally, we asked for hopeful expectations of a “church / religious community that welcomes all people in an appreciative and accepting way—and accepts them as they are and feel” (item z8).

All of these items were scored on a 5-point scale from disagreement to agreement (0—does not apply at all; 1—does not truly apply; 2—don’t know (neither yes nor no); 3—applies quite a bit; and 4—applies very much). The scores can be referred to as a 100% level (transformed scale score). Scores > 60% indicate higher agreement, scores between 40 and 60 indifference, and scores < 40 disagreement.

#### Spiritual Dryness

The perception of phases of spiritual dryness was measured with the 6-item *Spiritual Dryness Scale* (SDS-6) which has good internal consistency (Cronbach’s *α* = 0.87) (Büssing et al., 2013). The statements refer to participants´ feelings that God is distant, that one’s prayers go unanswered, as well as the feeling of being “spiritually empty” or not able to give anymore (in terms of spiritual exhaustion) and being abandoned by God. The response options on a Likert scale were “not at all” (1), “rarely” (2), “occasionally” (3), “fairly often” (4), and “regularly” (5). The SDS scores are the mean scores and represent the perceived lack/shortage. The internal consistency of the scale is good in this sample, too (Cronbach´s alpha = 0.86).

#### Religious Practices and Religious Trust

The frequency of private prayers and church attendance was assessed as 0—never; 1—seldom; 2—often; and 3—regularly. Religious trust was assessed by the item “My faith is a firm hold even in difficult times” (item a37) from the ASP questionnaire. It is scored on a 5-point scale from disagreement (0) to agreement (4).

#### Psychological Well-being

Psychological well-being was assessed with the 5-item *WHO-Five Well-being Index* (WHO-5) (Bech et al., 2003). The representative items are “I have felt cheerful and in good spirits” or “My daily life has been filled with things that interest me.” The intensity of feelings refers to the last 2 weeks and was scored with a 6-step grading scale ranging from “at no time” (0) to “all the time” (5). Scores < 13 may indicate low well-being or even depressive states. The internal consistency of the scale is good in this sample (Cronbach´s alpha = 0.83).

#### Life Satisfaction

Participants´ life satisfaction was measured using the Brief Multidimensional Life Satisfaction Scale (BMLSS) (Büssing et al., 2009). The items of the BMLSS address intrinsic (oneself and life in general), social (friendships and family life), external (work and living situation), prospective dimensions (financial situation and future prospects), and health (health situation and abilities to deal with daily life concerns) of life satisfaction as a multifaceted construct. The internal consistency of the instrument was found to be good in the validation study (Cronbach’s alpha = 0.87) and in this study, too (Cronbach’s alpha = 0.85).

The BMLSS was supplemented by an additional module addressing perceived satisfaction with either acceptance or support by different groups (Büssing et al., 2017). Here, we asked for satisfaction with the acceptance and also with the support as LGBTQI + by friends and local faith communities. These four items are the factor Support Satisfaction (Cronbach´s alpha = .80) with its sub-factors, (1) Support by Friends (Cronbach´s alpha = .91), and (2) Support by Church Community (Cronbach´s alpha = .90).

All items were introduced by the phrase “I would describe my satisfaction with … as ….” Scoring ranges from very dissatisfied (0) to very satisfied (6). The life satisfaction sum score was referred to as a 100% level (transformed scale score).

### Statistical Analyses

Descriptive statistics are presented as frequencies for categorical variables and as means (± standard deviation, SD) for numerical variables. Reliability analyses (Cronbach´s alpha), one-way analysis of variance (ANOVA) on ranks (Kruskal–Wallis test for non-normally distributed variables), as well as first-order correlations (Spearman rho) and linear regression analyses with stepwise variable selection method were computed with SPSS 28.0. Mediation analyses followed the methods described by Hayes (2017). In this procedure, both the direct effect and mediation effect are evaluated.

Given the exploratory character of this study and the various tested variables, we set a stricter significance level at *p* < 0.01. Concerning classifying the strength of the observed correlations, we adjusted the thresholds to *r* > 0.5 as a strong correlation, an r between 0.3 and 0.5 as a moderate correlation, an r between 0.2 and 0.3 as a weak correlation, and *r* < 0.2 as negligible or no correlation. For ANOVA, *η*^2^ values < 0.06 are considered as small effects, between 0.06 and 0.14 as moderate, and > 0.14 as strong.

## Results

### Description of Participants

Initially, 545 people started the survey. However, 128 of them (24%) provided only rudimentary information, so they were excluded from the analyses as “non-responder.” Both groups did not significantly differ in terms of their age, gender identity, or religious affiliation, but differed in sexual orientation. The proportion of people with a homosexual orientation was significantly higher in the group of “responder” as compared to the “non-responder” (59% vs. 24%; *p* < 0.001, Pearson Chi^2^).

The mean age of the 417 responders is 43 ± 16 [18–83] years. The majority regard themselves as female (45%) or male (46%), while 6% stated genderqueer/non-binary and 27 people trans*. Within the trans* group, three stated as gender identity female, six male, eight genderqueer/non-binary, and the remaining 10 as trans* without a further gender identification. For statistical analyses, their self-ascribed gender identity was chosen, and thus, 2% were categorized as trans* without a further gender identification (Table 1). Most participants regarded themselves as homosexual (59%), 17% bi/pansexual, 19% heterosexual, and 5% stated “other” as sexual orientation (Table 1). About 60% live with a permanent partner, 40% without.

**Table 1:** Description of participants (*N* = 417)

	*n*	%	Mean ± SD
Age (years)	407		43.1 ± 16.1 [18–83]
Gender identity	415	100	
Female	187	44.8	
Male	193	46.3	
Genderqueer/non-binary	27	6.5	
Trans*	10	2.4	
Sexual orientation	415	100	
Heterosexual	77	18,6	
Bi-/pansexual	71	17.1	
Homosexual	245	59.0	
Other	22	5.3	
Living with a permanent partner	363	100	
Yes	219	60.3	
No	144	39.7	
Religious affiliation	414	100	
Catholic	151	36.5	
Protestant	109	26.1	
A Free Churches	61	14.7	
Seventh-day Adventist	53	12.8	
Other denominations	4	1.0	
Non/not any longer	37	8.9	
Visibility in church	399	100	
As priests, pastors, deacons, and religious brothers and sisters	57	14.3	
As non-ordained pastoral workers, religion teachers	62	15.5	
As students preparing for a job in the church	62	15.5	
Other positions/professions not related to the church	218	54.6	
Frequency of praying	404		1.6 ± 0.9 [0–3]
Frequency of church attendance	409		1.7 ± 1.0 [0–3]
Trust as a strong hold in difficult times (A37)	394		3.2 ± 1.1 [0–4]
Perception of spiritual dryness (SDS item 3)	382	100	
Not at all	57	14.9	
Rarely	106	27.7	
Occasionally	140	36.6	
Fairly often	56	14.7	
Regularly	23	6.0	
Don´t believe in God any longer	403	100	0.6 ± 1.1 [0–4]
Agreement	32	7.9	
Indifference	36	8.9	
Disagreement	335	83.2	
Leaving the church	404	100	
No	275	68.1	
I consider it	69	17.1	
Yes	60	14.9	
Quality of life indicators			
Psychological well-being (WHO-5)	410		54.3 ± 19.2 [0–100]
Life satisfaction (BMLSS-10)	410		71.3 ± 15.9 [0–100]
Satisfaction with friends (BMLSS-Support)	376		82.4 ± 21.3 [0–100]
Satisfaction with church community (BMLSS-Support)	370		48.4 ± 31.5 [0–100]
Spiritual dryness (SDS-6)	381		2.4 ± 0.9 [1–5]

*Not all responded to all items

Among them, 37% were Catholics, 26% were Protestants, 15% were from Protestant Free Churches, 13% were Seventh-day Adventist (as one of the larger Protestant Free Churches), 1% from other denominations, and 9% had none (any longer).

About 21% experienced phases of spiritual dryness often or even regularly. Further, 8% stated that they do not believe in God any longer, and 9% are indifferent; 15% stated that they already have left the church, 17% are considering it, and 68% disagree.

Several of the participants have visibility in their churches as they are either priests/pastors/deacons or religious brothers or nuns (15%), non-ordained pastoral workers or teachers of religion (16%), and 16% are preparing themselves for a job in the church.

### Satisfaction with Acceptance and Support

Within the whole sample, most feel accepted (85%) and supported (79%) by their direct friends as LGBTQI+. However, only 28% are satisfied with the acceptance as LGBTQI+ by their local religious community (41% are dissatisfied and 17% are undecided), and 31% are satisfied with their local religious community's support (46% are dissatisfied and 23% are undecided). The discrepancy between their satisfaction with (emotionally closer) friends and with the local religious community is striking.

Satisfaction with acceptance and support by friends or the church community was not significantly different concerning gender identity (Table 2). However, the generally high support satisfaction by friends was highest in participants with homosexual orientation and lowest in heterosexual participants. The effect size is moderate (*η*^2^ = 0.117). The overall low satisfaction with the acceptance and support by the church community was not significantly different in the four groups of sexual orientation.

**Table 2:** Satisfaction, perception of discrimination, and loss of faith in different subsets

	Satisfaction with acceptance/support by friends	Satisfaction with acceptance/support by the local church community	Perception of discrimination	Loss of faith because of experiences in church	Self-acceptance
Range	0–100	0–100	0–100	0–100	0–100
*All participants*					
Mean	82.31	48.47	48.01	16.33	78.86
SD	21.36	31.56	32.80	21.84	20.91
Gender identity					
*Female*					
Mean	80.62	47.02	48.57	17.01	78.26
SD	21.68	27.48	33.50	22.36	21.16
*Male*					
Mean	84.76	51.10	44.31	14.17	80.19
SD	19.63	33.24	32.11	20.80	21.08
*Gender queer/non-binary*					
Mean	77.61	41.02	64.13	20.29	79.89
SD	26.42	40.33	29.27	22.87	17.16
Trans*					
Mean	78.32	43.71	65.18	29.76	66.96
SD	27.93	37.10	27.38	23.05	18.74
Kruskal–Wallis H	4.05	3.06	11.55	8.60	8.30
p value	n.s	n.s	0.009	0.035	0.040
Eta^2^ value	0.013	0.008	0.030	0.021	0.014
Sexual orientation					
*Heterosexual*					
Mean	65.92	51.18	18.55	15.33	77.39
SD	23.51	24.01	27.09	21.83	21.95
*Bi-/pansexual*					
Mean	77.96	46.81	55.28	17.31	78.17
SD	22.28	28.06	29.02	23.06	18.86
*Homosexual*					
Mean	87.13	49.35	51.84	15.99	79.96
SD	18.50	33.28	31.23	21.67	21.24
*Other*					
Mean	82.68	39.80	68.18	19.70	75.00
SD	20.01	37.68	29.56	21.45	17.68
Kruskal–Wallis H	41.46	0.70	57.81	0.28	2.00
*p* value	< 0.001	n.s	< 0.001	n.s	n.s
*η*^2^ value	0.117	0.006	0.166	0.002	0.005
Religious denomination					
*Catholic*					
Mean	82.41	47.56	50.08	13.36	80.83
SD	20.09	29.60	31.12	19.38	21.05
*Protestant*					
Mean	87.94	63.83	33.29	9.78	77.22
SD	17.52	29.66	32.30	16.89	20.88
*Free church*					
Mean	78.73	43.21	61.89	19.13	78.28
SD	23.43	30.76	28.54	21.64	19.75
*SDA*					
Mean	76.12	26.63	57.00	16.93	76.79
SD	27.48	26.36	31.36	20.20	18.22
*None (anymore)*					
Mean	79.54	40.46	49.17	46.70	82.20
SD	19.93	29.68	34.88	24.38	23.18
Kruskal–Wallis H	2.94	26.86	4.60	26.28	0.62
*p* value	n.s	< 0.001	n.s	< 0.001	n.s
*η*^2^ value	0.035	0.135	0.093	0.185	0.009

Concerning the underlying religious communities, the satisfaction with friends did not significantly differ (Table 2). The satisfaction with the acceptance of LGBTQI+ and support by the local religious community was lowest in SDA and highest in Protestants. This difference is moderate (*η*^2^ = 0.135).

Whether the participants have “visible” duties in the church or not has no influence on their acceptance and support satisfaction, perception of discrimination, or self-acceptance, but on loss of faith which was lowest in those with visibility (*η*^2^ = 0.004, *p* = 0.002) (data not shown).

### Perceptions and Experiences of the Respondents in the Parish

Participants’ perceptions and experiences in their parish are further differentiated (Table 3). For 36%, hardly anyone in the religious community knows anything about their GI/SO. As many as 42% feel accepted in their community regarding their GI/SO. Only 9% have difficulties reconciling their GI/SO with their belief.

**Table 3:** Experiences of acceptance in church, trust in God, and self-acceptance

Item ID	Statement	Does not apply at all (%)	Does not really apply (%)	I don´t know (neither yes nor no) (%)	Applies quite well (%)	Definitely applies (%)
**z1**	I feel discriminated against by representatives of the church/religious communities because of my gender identity or my sexual orientation	17.7	15.4	13.7	28.6	24.6
**z2**	I just don't feel comfortable in my church/religious community anymore because of my gender identity or sexual orientation	35.8	16.2	17.5	14.5	16.0
z3	Very few people in my church/community know about my gender identity or sexual orientation	33.6	18.6	12.0	15.0	20.6
z4	I feel accepted in my church/community regarding my gender identity or sexual orientation	15.9	18.5	23.3	22.8	19.5
z5	I have trouble aligning my gender identity or sexual orientation with my beliefs	65.6	18.6	7.1	6.6	2.0
**z6**	Because of my experiences as an LGBTQI* person with people in the churches/religious community, I left the church	71.4	4.3	10.7	6.4	7.2
**z7**	Because of my experiences as an LGBTQI* person with people in the churches/religious community, I lost my faith	65.3	15.6	8.5	9.0	1.6
**a0**	I don't believe in God anymore	72.7	10.6	9.1	3.8	3.8
a1	I am sure that God loves and accepts me just the way I am	2.0	0.5	4.6	17.9	75.0
a37	My faith is a firm hold even in difficult times	3.8	4.3	13.5	29.2	49.2
z8	I wish for a church/religious community that welcomes all people in an appreciative and accepting way—and accepts them as they are and feel	0.5	0.8	3.0	6.0	89.7
R10	I have found a positive attitude toward myself	0.8	3.3	10.1	39.9	46.0
R1	All in all, I'm happy with myself	1.8	4.8	14.6	43.8	35.0

However, 53% see themselves discriminated against by representatives of the church/religious communities because of their GI/SO, and 31% no longer feel comfortable in their community because of their GI/SO. About 14% stated that they had left the church because of their negative experiences with representatives of churches or religious communities, 11% said that they had lost their faith because of these experiences, and 8% do not believe in God (anymore) (Table 3).

Characteristic free text statements substantiate these empirical findings:

“In my local community, there is almost no problem. However, the three or four conservative parishioners are enough to make me feel very uncomfortable. Since they now feel strengthened by Rome and by some bishops, nothing will change. This burdens me so much that I have given up my job, which I have been doing for almost 30 years, and have made my way to freedom. I can no longer celebrate church service with these people and have thus lost my spiritual home.” (#114, 52, m, Catholic)

“I'm very sad that most of my community doesn't accept me with my trans* (non-binary) identity. I'm still out there as binary trans*, outed as a boy, and unfortunately I'm not accepted.” (#360, -, t; SDA)

“After many fights and discriminatory experiences, I just don't have the words anymore. I used to work full-time as a youth advisor and deacon, but due to the anti-attitude of many Christians I gave up this job and can no longer do anything with faith and church.” (#214, 34, f, Protestant)

“After my coming out 20 years ago, I had very bad experiences there. I was discriminated against until I resigned. Today I have found my home in a gay-friendly Methodist church.” (#347, 50, m, Free church)

“I've been insulted by my church as ‘possessed by the devil,’ and as an ‘eyesore.’ The other church to which I switched only wanted me unofficially, but not officially. That's why I left the church, I'm still an Adventist at heart.” (#64, 52, m, SDA)

“I didn't want to leave, I was kicked out. In my new church, however, I miss the feeling of home.” (#234, 37, m, Free church)

For 78%, their faith remains a firm hold even in difficult times. Nevertheless, 96% want a church/religious community that is appreciative and accepting of all people—and accepts them as they are and feel, and 93% are sure that God loves and accepts them as they are.

“My beliefs have changed a lot in the last few years. I firmly believe that in the future I will find a place in a church/community where I can live my faith fully and still be myself.” (#541, 29, f, Catholic)

“I believe (since childhood) that my Creator did not create me defective. But that I am what He wanted that I am right, and that it is my job to live it, despite all the difficulties that my fellow creatures put in my way. This trust in God gave me the strength to go my way.” (#461, 66, t, Protestant)

### Perceptions of Acceptance and Discrimination, and Loss of faith and Self-Acceptance

As shown in Table 2, discrimination was perceived significantly stronger in participants with a genderqueer/non-binary identity than in participants with a binary gender identity (with weak effect size) and was lowest in participants with a heterosexual orientation as compared to other orientations (with strong effect size). However, these perceptions were higher in participants from Free Churches and lower in Protestants (with moderate effect size), but not significantly different.

The consequence of these experiences can be a loss of faith because of such negative experiences in their church. This loss of faith was not significantly different for sexual orientation and in trend only for gender identity (Table 2). However, those who stated that they don´t belong to a church community anymore had the highest loss scores as compared to those with a church affiliation (with a strong effect size).

Participants´ self-acceptance was high, and the scores did not significantly differ for gender identity, sexual orientation, or religious denomination (Table 2).

### Associations Between Acceptance, Loss of Faith, and Quality of Life Indicators

Satisfaction with the acceptance as LGBTQI+ and support by friends was moderately related to general life satisfaction and self-acceptance and weakly only to psychological well-being (Table 4). In contrast, satisfaction with the acceptance and support by the church community was strongly negatively related to the perception of discrimination. Further, both the satisfaction with the church and the perception of discrimination were moderately associated with spiritual dryness and with loss of faith because of the negative experiences in church, and weakly related to general life satisfaction and well-being (Table 4).

**Table 4:** Correlation analyses

	Satisfaction with acceptance/support by Friends	Satisfaction with acceptance/support by local church community	Perception of discrimination	Loss of faith because of experiences in church	Self-acceptance
Age (years)	.075	.195**	−.264**	.107	.166**
Satisfaction with friends	1.000				
Satisfaction with the church community	.356**	1.000			
Perception of discrimination	−.185**	−.618**	1.000		
Loss of faith because of experiences in church	−.170**	−.323**	.380**	1.000	
Self-acceptance	.337**	.175**	−.177**	−.196**	1.000
Well-being (WHO-5)	.217**	.227**	−.213**	−.119	.455**
Life satisfaction (BMLSS-10)	.348**	.267**	−.273**	−.177**	.594**
God loves and accepts me just the way I am (A1)	.164**	.155**	−.087	−.480**	.387**
Faith as a firm hold even in difficult times (A37)	.131	.229**	−.204**	−.581**	.308**
Spiritual dryness (SDS-6)	−.271**	−.340**	.340**	.386**	−.428**

**p < 0.001 (Spearman rho)

Moderate and strong correlations are highlighted in bold

The perception that one has lost faith because of the negative experiences in the church was strongly correlated with (low) religious trust and moderately with spiritual dryness. Its association with well-being and life satisfaction was marginal only.

The ability to accept oneself as one is and feels was strongly related to life satisfaction, and moderately to psychological well-being, religious trust, and (lower) spiritual dryness (Table 4).

Satisfaction with acceptance/support by the local church community was marginally related to age, but not to satisfaction with friends, while the perception of discrimination was weakly negatively related to age, but not loss of faith (Table 4).

### Influence on Life Satisfaction and Well-Being

Are these negative perceptions associated with life satisfaction and well-being (which are strongly interrelated, *r* = 0.66, but conceptually different)? To answer this, regression analyses with the following influencing variables derived from the theoretical framework (Fig. 1) were performed:

1. Satisfaction with friends and with the local church community
2. Perception of discrimination and loss of faith because of negative experiences in the church
3. Self-acceptance and sense of acceptance by God
4. Religious trust (“Faith as a firm hold”) and spiritual dryness

As shown in Table 5, life satisfaction as a dependent variable was significantly influenced by self-acceptance and (low) spiritual dryness, and also by living in a stable partnership and satisfaction with the acceptance/support by friends. Religious trust was a negative predictor in trend, while religious practices were a positive predictor in trend. Perception of discrimination, loss of faith, being accepted and loved by God, and satisfaction with the church community had no significant independent influence in this model. Further, gender identity, sexual orientation, and age had no significant influence. This model explains 54% of the variance.

**Table 5:** Predictors of life satisfaction (regression analyses)

Dependent variable: Life satisfaction (BMLSS-10) Model 1: *F* = 17.2, *p* < 0.001; *R*^2^ = .54	Beta	*T*	*p*
(constant)		6.298	< .001
Age	−.013	−.262	.794
Gender identity: female	−.023	−.154	.878
Gender identity: male	.054	.363	.717
Gender identity: genderqueer/non-binary	−.057	−.685	.494
Gender identity: trans*	−.055	−.973	.332
Sexual orientation: heterosexual	.013	.180	.857
Sexual orientation: bisexual	−.022	−.310	.757
Sexual orientation: pansexual	.065	1.425	.155
Sexual orientation: homosexual	−.051	−.581	.562
Living with a partner	**.195**	**4.424**	** < .001**
Satisfaction with friends	**.189**	**3.730**	** < .001**
Satisfaction with the local church community	.034	.589	.556
I am sure that God loves and accepts me just the way I am (A1)	−.084	−1.529	.127
Religious trust (A37)	−.138	−2.333	.020
Religious practices (SpREUK-P)	.094	1.902	.058
Perception of discrimination	−.052	−.861	.390
Loss of faith because of experiences in church	.051	.883	.378
Spiritual dryness (SDS-6)	−**.304**	−**5.879**	** < .001**
Self-acceptance	**.411**	**8.043**	** < .001**

Psychological well-being as a dependent variable was similarly influenced by self-acceptance (Beta = 0.34, *T* = 5.49, *p* < 0.001) and spiritual dryness (Beta = 0.21, *T* = −3.43, *p* < 0.001), and in trend by living with a partner (Beta = 0.13, *T* = 2.46, *p* = 0.014), while the other variables had no significant influence in this model that explains 34% of the variance (data not shown).

In both models, self-acceptance and spiritual dryness were the main relevant predictors of life satisfaction and well-being, while satisfaction with acceptance/support by the local church community, perception of discrimination, and loss of faith because of experiences in the church had no independent influence. Stepwise regression analyses revealed that the best predictors of spiritual dryness as a dependent variable were (low) religious trust, perception of discrimination, (low) self-acceptance, lacking a stable partnership, loss of faith, and lower age (Table 6). These six variables would explain 36% of the variance. Without significant influence in this model were gender identity, sexual orientation, support satisfaction (friends or local church community), feeling loved and accepted by God, and religious practices.

**Table 6:** Predictors of life satisfaction (stepwise regression analyses)

Dependent variable: Spiritual dryness (SDS-6) Model 6: *F* = 27.9, *p* < 0.001; *R*^2^ = .36	Beta	*T*	*p*
(constant)		16.158	< .001
Faith as a firm hold even in difficult times (A37)	−.179	−3.049	.003
Perception of discrimination	.158	3.030	.003
Self-acceptance	−.240	−4.826	< .001
Living with a partner	−.138	−2.839	.005
Loss of faith because of experiences in church	.187	3.126	.002
Age	−.148	−2.980	.003

**Fig. 2:**
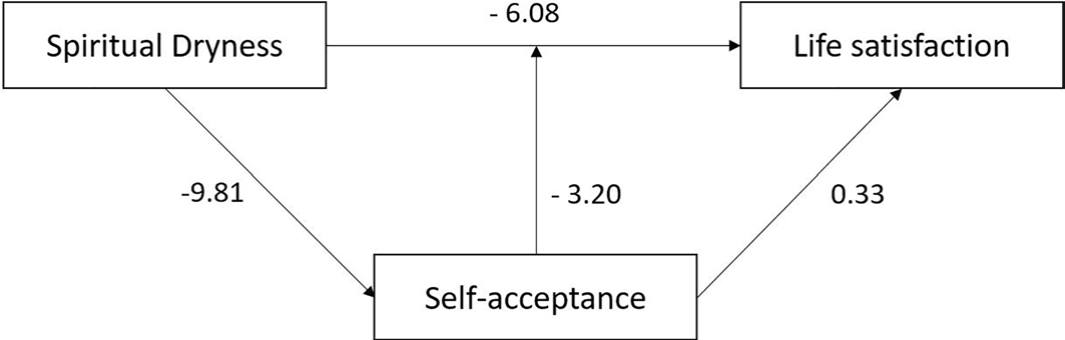
Mediation analysis with self-acceptance as a mediator between spiritual dryness and life satisfaction. Depicted are standardized beta values with *p*-values

As self-acceptance plays a crucial role in the interaction of spiritual dryness and quality of life indicators, we run a mediation analysis to determine the direct and indirect effects of these variables. In this analysis, both the direct effects of spiritual dryness and self-acceptance on life satisfaction were assessed, and also, the indirect path from spiritual dryness on life satisfaction mediated by self-acceptance. As shown in Fig. 2, life satisfaction is explained by spiritual dryness (*β* = −6.08, *p* < 0.0001) and by self-acceptance (*β* = 0.33, *p* < 0.0001) directly, having approximately 41% of the variance explained (*R*^2^ = 0.41). The relationship between spiritual dryness and life satisfaction is further mediated by self-acceptance (*β* = −3.20, *p* < 0.0001) and represents 35% of the total effect; the total effect from spiritual dryness on life satisfaction is *β* = −9.27 (*p* < 0.0001). Similarly, psychological well-being (WHO-5) is explained by spiritual dryness directly (*β* = −1.61, *p* < 0.0001) and by self-acceptance (*β* = 0.08, *p* < 0.0001), having approximately 26% of the variance explained (*R*^2^ = 0.26). Also, the relationship between spiritual dryness and well-being is mediated by self-acceptance (*β* = −0.74, *p* < 0.0001) and represents 32% of the total effect; the total effect from spiritual dryness on well-being is *β* = −2.34 (*p* < 0.0001).

## Discussion

The findings from this explorative study can be summarized as follows: People who belong to the LGBTQI+ community experience both acceptance and exclusion in their churches—up to clear discrimination and requests to leave the community, depending on the church’s and parish’s position and policy on LGBTQI+ issues. Some "hide" themselves to avoid confrontations and painful discussions about their status in the community. These negative reactions influence the individual’s social, emotional, and spiritual domain of life satisfaction. The fact that loss of faith, as observed by some of them, was caused by the behavior of church leaders and parishioners is in itself serious.

Many of the respondents still have the hope that the positions of their church and the attitudes and behaviors of church representatives could change in the long term—which is also expressed in the desire of the vast majority for a church/community that is appreciative and accepting of all people. This might be seen as too idealistic, but it nevertheless indicates their longing for a secure haven in the church. Some are not leaving their church as they also experience support from community members. Also, in a qualitative study by Brennan-Ing et al. (2013), LGBT adults reported feelings of support from their congregations, particularly provided by pastors and religious leaders. In our study, most participants remain sure that God loves and accepts them just the way they are. This could be interpreted as a matter of hope that their church will become or remain their spiritual home, which is substantiated by the fact that some did find more accepting churches.

This is exactly what the German campaign "Out in Church—For a church without fear” was based on in January 2022, which caused a stir when 125 employees from the Catholic Church came out as lesbian, gay, bi, trans*, inter, queer, and non-binary (Brinkschröder et al., 2022). This brought them a lot of sympathy and led to a rethinking in the German branch of the Catholic Church, too. Our study found that the visibility of LGBTQI+ persons who work within and for the church as priests, pastors, pastoral workers, and teachers is still an issue, supporting Hancocks et al.’s (2008) findings on LGB chaplains as “refugees.” However, the question remains whether all the different churches with their diversity of positions, statements, and members can really be a home for people with different identities. The Catholic priest and Franciscan Norbert Lammers and the former priest Stefan Diefenbach plead for exactly this from a Franciscan point of view (Lammers & Diefenbach, 2023). Also, Adventist pastor Alicia Johnston answers this with a clear Yes and is committed to an “affirmative theology,” which is also God’s promise based on the Bible (Johnston, 2022). The protestant pastoral care expert and university chaplain Kerstin Söderblom conceptualizes pastoral care specifically directed at LGBTQI+ people as “queer sensitive pastoral care” encouraging and empowering LGBTQ+ persons to experience acceptance by God, faith community, and self (Söderblom, 2023).

The findings of this study reveal that the experiences were significantly different in the different churches. In particular, satisfaction with the acceptance of LGBTQI+ and support by the local church community was higher in Protestant churches and lowest in SDA, while perception of discrimination was highest in Protestant Free Churches with their often stricter positions. This was underlined in the free text statements. Also in the USA, mainline Protestant churches seem to be more accepting than the Catholic church or Evangelical churches (Barringer, 2020). Saunders et al. (2023) argued that even when there are trends to liberalization “anti-LGBTQ attitudes remain widespread within many religious organizations, even those that aim to be more inclusive institutions” (p. 13).

A further interesting detail is that the perception of discrimination was weakly and inversely related to age. This means that younger participants experience it much more strongly than older participants who may have found ways to cope with these feelings and to endure (or ignore) the negative experiences. We do not assume that this is a matter of religious “immaturity” but higher sensitivity and higher expectations at younger ages. This could also be a matter of self-acceptance, which was strongly related to life satisfaction and moderately to psychological well-being (and was their best predictor anyway), and of certainty that God loves and accepts them just the way they are, but only marginally related to age (*r* = 0.166, *p* < 0.001).

Those who can accept themselves experience less frequent phases of Spiritual dryness, which is a specific form of spiritual crisis where God is perceived as distant and non-responding (Büssing & Dienberg., 2019, 2021). It is not surprising that the experience of spiritual dryness is inversely related to the acceptance/support by the local church community and positively to the perception of discrimination by church members. In this study, spiritual dryness furthermore is a relevant negative predictor of life satisfaction and psychological well-being. Also, in qualitative interviews with religious brothers and sisters, conflicts with close community members were identified as triggers of spiritual dryness (Büssing et al., 2020). This means that emotional conflicts within the church community can affect the relation towards God, with the risk that they lose their faith in the long run when they assume they are neither wanted by their church nor by God. Further, the spiritual dryness of Catholic priests, who have to live in celibacy, was predicted by a lack of transcendence perception and low life satisfaction, on the one hand, and by “problems with sexuality” (and accepting their sexual orientation) and an inability to be alone (independently from their social network), on the other hand (Baumann et al., 2017). This indicates that the conditions of the relational life toward others and their own sexual identity can influence the relation toward God, too. Whether this could be seen in the context of the findings of Lapinsky & McKiernan (2013) that people with LGB identity who have left their church “viewed God as hostile” remains unclear. At least, we can state that in our study, only a very small group stated feelings that “God has abandoned me completely” (*n* = 16). Referring to the intention to leave the church, 10% of those who consider leaving their church experience this “abandonment” often to regularly, and 10% of those who have already left their church, while these feelings were reported by only 2% of those who do not consider leaving their church (*p* < 0.001; Chi^2^).

In this study, self-acceptance was identified as the best predictor of participants´ life satisfaction and psychological well-being. As stated above, this aspect of inner strength may buffer negative statements of others (MacInnes, 2006) and was found to be a protective factor in LGBQ+ people stabilizing their psychological well-being (Camp et al., 2020). In this study, self-acceptance did not differ for GI/SO, which is in contrast with the findings by Matud et al. (2019) who reported higher scores in men than in women. Surprisingly, both loss of faith because of negative experiences in the church and satisfaction with the acceptance and support by the church community had a marginal but negative influence on self-acceptance. Whether this means that those with lower self-acceptance are more satisfied with the support and acceptance by the church community than those with high self-acceptance, who may not expect such an external acceptance, is unclear. Although the effects are only small, this could further mean that the negative experiences with a loss of faith could trigger an attitude of “resistance” to stand their ground and identity and make themselves more independent from the often critical views of others.

According to the minority stress concept of Meyer (2003), the objective distal stressors (i.e., objective situations and conditions) can be independent of personal identifications, while the proximal stress processes refer to subjective perceptions and their interpretations. The fact that self-acceptance was a relevant buffer of spiritual dryness, and both were the best predictors of life satisfaction and well-being is an important finding. This means that it is not gender identity or sexual orientation per se that has an impact on life satisfaction; these are (with exceptions) quite similar across the entire group. The perception of discrimination and exclusion is nevertheless associated with lower well-being among the participants, but it is not a significant predictor. Self-acceptance and spiritual dryness are of greater importance and override the effect of the perception of discrimination. One is a stabilizing resource and a mediator of the link between spiritual dryness and life satisfaction, and the other is a possible negative of the experienced outcome in terms of religious struggle.

According to Ellison and Lee (2010), there are different types of spiritual struggles, (1) troubled relationships with God (here perception of spiritual dryness in terms of God); (2) negative social encounters (here perception of low acceptance and support by the local church community and feeling excluded or even discriminated), and (3) intrapsychic processes (which could be attributed to self-acceptance in this study). In their study, moderate interactions between troubled relationships with God and psychological distress were found, but only marginal correlations between negative interactions and troubled relations with God or psychological distress (Ellison & Lee, 2010). In our study, we see similarly weak associations between satisfaction with acceptance/support by the local church community or perception of discrimination with well-being and life satisfaction, but a stronger impact of spiritual dryness on these quality of life-associated variables.

**Fig. 3:**
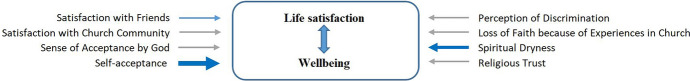
Approved model of positive and negative influences on life satisfaction and wellbeing. The thickness of the arrows represents the strength of the association

To the theoretical framework of this study, we can state that self-acceptance and (low) spiritual dryness were the best predictors of both participants´ life satisfaction and well-being, with a further weak influence of satisfaction with the support by friends (Fig. 3). Dissatisfaction with the acceptance as LGBTQI+ by the local church community, the perception of discrimination, and subsequent loss of faith were experienced and have their major role, but from a statistical point of view, their relevance is secondary to the dominating role of self-acceptance.

## Limitations

Findings from cross-sectional studies do not allow causal interpretations. Therefore, we have used the free text comments to substantiate the observations and interpretations.

We do not assume that our findings are representative of all LGBTQI+ people in Germany, as we surely did not reach all of them. As our routes of recruitment focused on church-related participants, we probably have reached particularly those who still have hope that the positions of the churches will be more accepting in the future, while those who have lost this hope and have left their church might not be reached by our routes. In fact, we received rather hostile emails from former Catholic priests who explained that they did not wish to participate to avoid secondary trauma.

Study participants have identified themselves as part of the LGBTQI+ community and made statements on their gender identity and sexual orientation. The fear of being identified by sharing anonymous information might have been higher than expected as several started the survey but did not provide relevant data or complete the full questionnaire. They had to be excluded from the analyses. Both groups did not differ significantly in terms of their gender identity, age, or religious affiliation, but for their sexual orientation, indicating that it is a sensitive topic.

## Conclusions

The interviewees experienced both acceptance and exclusion, including discrimination and requests to leave their church. This discrimination in the church is differentially experienced depending on participants´ GI/SO, which had no relevant influence on their self-acceptance or their generally low satisfaction with the acceptance and support by the local church community. Nevertheless, the negative perceptions can trigger or aggravate phases of spiritual dryness which can finally result in loss of faith. This specific form of religious struggle is a negative predictor of participants´ well-being and life satisfaction. As the link between spiritual dryness and life satisfaction (and well-being) is mediated by self-acceptance, this intrapersonal resource could be supported by a church that accepts people as they are and how they feel. In fact, most participants still hope that the often negative attitudes and positions of representatives of the churches will change in the long term, indicating hope that they expect the church to be a secure haven.

## Data Availability

Not applicable.

## Footnotes

1 The acronym LGBTQI+ is currently used as an umbrella term that attempts to embrace different sexualities and gender identities. It stands for lesbian, gay, bisexual, transgender, queer, intersex, and a variety of other identifications and identities. For our study, we will used this term, while we cite variant versions as they were stated.
